# Profile of Inflammation-associated genes during Hepatic Differentiation of Human Pluripotent Stem Cells

**DOI:** 10.1016/j.dib.2015.10.023

**Published:** 2015-11-09

**Authors:** Joseph Ignatius Irudayam, Deisy Contreras, Lindsay Spurka, Songyang Ren, Vidhya Kanagavel, Arunachalam Ramaiah, Alagappan Annamalai, Samuel W. French, Andrew S. Klein, Vincent Funari, Vaithilingaraja Arumugaswami

**Affiliations:** a Board of Governors Regenerative Medicine Institute, Cedars-Sinai Medical Center, Los Angeles, CA 90048, USA; b Cedars-Sinai Genomics Core, Medical Genetics Institute, Cedars-Sinai Medical Center, Los Angeles, CA 90048, USA; c Centre for Infectious Disease Research, Indian Institute of Science, Bangalore, Karnataka 560012, India; d Hindustan Genomics Institute, SVA Medical Center, Kadayam, Tamil Nadu 627415, India; e Department of Surgery, Cedars-Sinai Medical Center, Los Angeles, CA 90048, USA; f Department of Pathology and Laboratory Medicine, University of California at Los Angeles, Los Angeles, CA 90095, USA; g Department of Surgery, University of California at Los Angeles, Los Angeles, CA 90095, USA

**Keywords:** pluripotent stem cells, hepatic cells, endoderm, hepatoblast, hepatocytes, differentiation, differential gene expression, inflammation, cytokines, sirtuin, SIRT1

## Abstract

Expression of genes associated with inflammation was analyzed during differentiation of human pluripotent stem cells (PSCs) to hepatic cells. Messenger RNA transcript profiles of differentiated endoderm (day 5), hepatoblast (day 15) and hepatocyte-like cells (day 21) were obtained by RNA sequencing analysis. When compared to endoderm cells an immature cell type, the hepatic cells (days 15 and 21) had significantly higher expression of acute phase protein genes including complement factors, coagulation factors, serum amyloid A and serpins. Furthermore, hepatic phase of cells expressed proinflammatory cytokines IL18 and IL32 as well as cytokine receptors IL18R1, IL1R1, IL1RAP, IL2RG, IL6R, IL6ST and IL10RB. These cells also produced CCL14, CCL15, and CXCL- 1, 2, 3, 16 and 17 chemokines. Endoderm cells had higher levels of chemokine receptors, CXCR4 and CXCR7, than that of hepatic cells. Sirtuin family of genes involved in aging, inflammation and metabolism were differentially regulated in endoderm and hepatic phase cells. Ligands and receptors of the tumor necrosis factor (TNF) family as well as downstream signaling factors TRAF2, TRAF4, FADD, NFKB1 and NFKBIB were differentially expressed during hepatic differentiation.

**Specifications Table**

**Table:** 

Subject area	*Biology*
More specific subject area	*Stem cell and Liver Biology*
Type of data	*Tables and figures (graphs)*
How data was acquired	*RNA sequencing analysis*
Data format	*Analyzed format*
Experimental factors	*Human pluripotent stem cells were induced to differentiate into hepatic cells using a three phase protocol with phase-specific cocktails of factors.*
Experimental features	*PSCs were subjected to a 21 day protocol for generating hepatocyte-like cells via endoderm induction (days 1 to 5), hepatic specification (days 6 to 15) and hepatic maturation (days 16 to 21) phases.*
Data source location	*RNA expression data was collected from stem cell differentiation experiments performed at Cedars-Sinai Medical Center, Los Angeles, USA.*
Data accessibility	*Data deposited to Gene Expression Omnibus (GEO) repository with an accession number GSE67848.*

**Value of the data**

• Expression profiles of inflammation-associated genes present during *in vitro* differentiation of endoderm and hepatic cells are provided.
• Stem cell-derived hepatic cells are being investigated for liver regeneration. The immune and inflammatory signatures of transplantable cells can influence the rate and severity of immune rejection.
• Endoderm phase of cells expressed lower levels of proinflammatory interleukins, interleukin receptors and chemokines than that of the hepatic phase cells.
• Therefore, the immature endoderm progenitor cells may elicit less immune response by the host.
• Priming of differentiated hepatic cells with immune modulatory and anti-inflammatory agents can help enhance engraftment and therapeutic potential.

**Data**

The main objective is to analyze the expression of inflammatory genes in hepatic cells differentiated from pluripotent stem cells. RNA sequencing analysis was performed on PSC-derived endoderm (day 5), hepatoblast (day 15) and hepatocyte-like cells (day 21). First, we examined the genes specific for hepatic function of the acute phase response (Fig. 1). Liver is the major source of acute phase proteins [1]. During acute phase of microbial infection or other injuries, the serum concentrations of these proteins change to mediate and regulate the inflammatory response [2], [3]. As expected, genes coding for acute phase proteins, including complement factors, coagulation factors, serum amyloid A1 (SAA1), alpha 2-macroglobulin (A2M), hepcidin antimicrobial peptide (HAMP), orosomucoid (ORM1 and ORM2), and alpha-1-antitrypsin (SERPINA1), were highly upregulated in the hepatic phase cells (days 15 and 21) compared to that of endoderm cells (Fig. 1).

Subsequently, we focused on genes involved in modulating cell homing and inflammatory responses. We observed that the hepatic cells had high levels of chemokines such as CCL14, CCL15, CXCL1, CXCL2, CXCL3, CXCL16 and CXCL17 (Fig. 2A). Chemokines are involved in recruitment of neutrophils, eosinophils, monocytes and T-cells to the site of injury [4], [5], [6]. This observation is important in the context of cell therapy and can be targeted for increasing the engraftment efficiency. On the other hand, chemokine receptors CXCR4 and CXCR7 were upregulated during the endoderm phase. CXCR4 is considered as a marker for endoderm phase and likely involve in cell migration during the embryonic gastrulation stage [7]. It is possible that the up-regulation of CXCL2, CXCL3, and CXCL16 could be explained by the existence of other endoderm/mesoderm lineage induced cells during differentiation. The multifaceted interaction of the growth factor cocktail, along with differentiating hepatic cells and accompanying minor mesendoderm cell population as well as damage-associated molecular pattern (DAMP) signals from dead cell debris in the culture can induce innate and inflammatory responses.

Furthermore, we have also observed differential expression of Sirtuin (SIRT) family of genes during hepatic differentiation. SIRT genes code for NAD-dependent protein deacetylases that are involved in aging, inflammation, differentiation, cancer and metabolism [8], [9], [10]. SIRT1 gene was upregulated at the endoderm phase, whereas SIRT2, SIRT3 and SIRT7 genes were induced at hepatic phase (Fig. 2B). SIRT1 has been shown to play anti-inflammatory role [11].

We also noted that once the cells differentiated to the hepatic lineage phase, the genes coding for proinflammatory cytokines and receptors were activated. The genes for IL18, IL32, IL18R1, IL1R1, IL1RAP, IL2RG, IL6R, IL6ST and IL10RB were upregulated in differentiated hepatic cells (Table 1). IL17 family of receptors showed differential expression during differentiation. Next, we analyzed genes participating in the TNF signaling pathway (Table 2). In the differentiated hepatic cells, TNF family ligands (TNFSF10, TNFSF12, and TNFAIP8L3), receptor (TNFRSF10B) and downstream signaling factors (NFKB1 and NFKBIB) were expressed at higher levels to that of endoderm cells. Interestingly, genes for number of TNF family receptors (TNFRSF11A, TNFRSF11B, TNFRSF19, and TNFRSF25) and adaptor signaling factors (TRAF2, TRAF4 and FADD) were down-regulated in hepatic cells. Caspase genes (CASP 4, 6 and 8) and other signaling pathway genes important for inflammation (MAPK1, MAPK13, IRF1 and NLRX1) also showed a trend of increased expression at the hepatic phase (Table 2). The up-regulation of TNFSF10 (TRAIL) and CASP4/8 indicates that during the final phase of hepatic differentiation, the supplemented factors HGF and OSM not only can promote maturation of hepatic cells but also select hepatic cells by inducing apoptosis of non-hepatic lineage cells. The major histocompatibility complex (MHC) genes, human leukocyte antigen (HLA) markers, exhibited differential regulation during the hepatic specification and maturation phases (Table 2). Detailed analysis, FPKM values of biological duplicates, *P*-values and means are given in Supplementary Table 1.

## 1 Experimental design, materials and methods

### 1.1 Cells and hepatic differentiation protocol

Human embryonic stem cell (hESC) line, WA09 (H9), was obtained from WiCell Research Institute, USA. The cell line was cultured using serum-free chemically-defined media, mTeSR1 (STEMCELL Technologies, Canada). We utilized a feeder free culture condition with a daily media change regimen. The cells were maintained at 37 °C with 5% CO_2_. We utilized a three phase protocol for hepatic differentiation of the pluripotent stem cell line as described previously [12], [13], [14]. The objective was to profile the transcriptome of endoderm (day 5), hepatoblast (day 15) and hepatocyte-like cells (day 21) derived from hESCs. The PSCs were induced to endoderm with Activin A (100 ng/ml, Peprotech Inc.), and Wnt 3A (40 ng/ml, R and D Systems) supplements in RPMI media (Life Technologies) for the first one day and the following four days with Activin A, VEGF (10 ng/ml), bFGF (10 ng/ml) and BMP4 (0.5 ng/ml) in serum-free defined (SFD) media. From day 6 onwards, the SFD media supplemented with VEGF (10 ng/ml), BMP4 (50 ng/ml), TGF-α (20 ng/ml), EGF (10 ng/ml), HGF (100 ng/ml), DMSO (1%, Sigma-Aldrich, St. Louis, MO), dexamethasone (1×10^−7^ M; Sigma-Aldrich), monothioglycerol (4.5×10^−4^ M), and ascorbic acid (50 μg/ml), was used. From day 12 onwards, TGF-α and BMP4 were removed from the cocktail. From day 16 onwards, for hepatocyte maturation, dexamethasone, HGF, and oncostatin M (20 ng/ml) were included in the SFD media. The differentiation was done in biological replicates and cells were harvested at indicated time points (day 5, day 15 and day 21 post-differentiation) for RNA isolation.

### 1.2 RNA sequencing and data analysis

A detailed description of RNA sequencing analysis, including RNA isolation; library preparation; quality assessment, filtering and alignment of RNA sequencing; gene expression filtering; and transcriptome analysis are provided in accompanying research paper by Ignatius Irudayam et al. [14]. Sequencing was performed on an Illumina HiSeq 2500 and obtained an average read depth of 26 million reads per sample. Quality control of raw illumina reads was done by RNASEQC [15]. TOPHAT program was used for aligning reads to the UCSC human reference genome (hg19) [16]. Reference-guided transcript assembly using a gene transfer file (GTF) from UCSC genes was used for calculating gene expression. For all samples, normalized quantification of reads as RPKM (# of Reads Per Kilobase of ORF per Million reads aligned) was calculated with Cufflinks [17] and subsequently was compiled into a gene counts table. For gene expression filtering, we utilized raw FPKM (Fragments Per kilobase of transcript per Million fragments mapped) values of over 1.1 to prevent overestimating gene expression differences between groups of samples and to permit downstream analyses. Raw FPKM values less than 1.1 were considered poorly measured. For calculating uniquely expressed genes, we used FPKM 5 as cut-off which represents 1 transcript copy per cell [18]. Genes with minimum FPKM value of 5 in one of the time points were included for downstream analysis. Data can be accessed form Gene Expression Omnibus (GEO) repository using accession number GSE67848.

## Figures and Tables

**Fig. 1:**
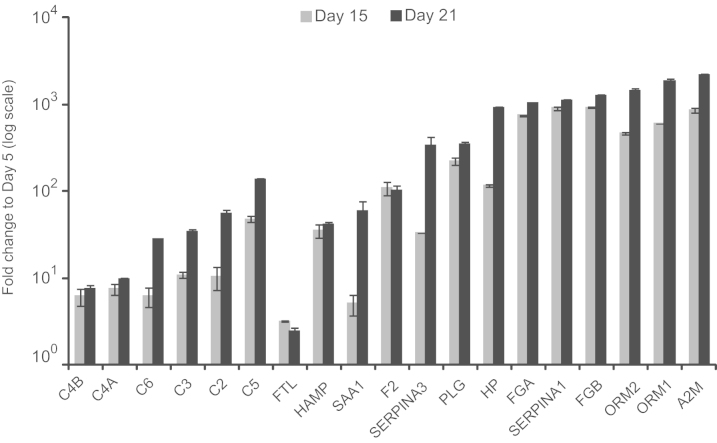
Expression of genes involved in acute phase response during day 15 and day 21 post-differentiation of PSCs**.** The absolute expression values of each time points (Day 15 and Day 21) were used for calculating fold change with that of Day 5. Mean values with standard deviation are shown in the graph.

**Fig. 2:**
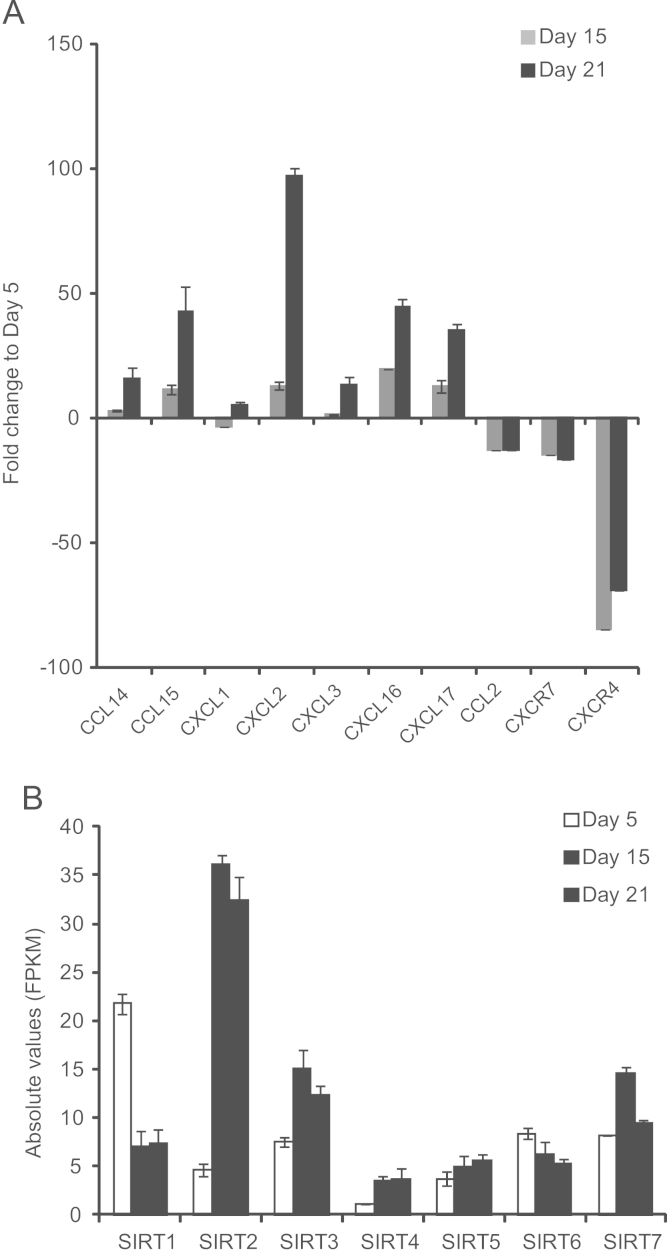
Differential expression of chemokine, chemokine receptor and sirtuin genes**.** (A) Mean values with standard deviation of fold change are depicted in the graph. Note that the receptor genes CXCR4 and CXCR7 were down-regulated in day 15 and day 21 hepatic cells. (B) Absolute expression values of sirtuin (SIRT) family of genes were shown in the graph with standard deviation.

**Table 1:** Differentially regulated cytokines and cytokine receptors.

**Feature ID**	**Gene ID**	**Fold change (Day 15 vs Day 5)**	**Fold change (Day 21 vs Day 5)**	***P*-value (Day 15 vs Day 5)**	***P*-value (Day 21 vs Day 5)**
ENSG00000150782.7	IL18	15.76	30.02	0.03	3.76E−05
ENSG00000008517.11	IL32	27.42	57.93	2.63E−03	0.12
ENSG00000196083.5	IL1RAP	39.58	56.85	4.39E−03	3.83E−03
ENSG00000160712.8	IL6R	32.12	26.58	6.22E−03	0.01
ENSG00000147168.7	IL2RG	35.63	23.98	0.03	0.17
ENSG00000134352.14	IL6ST	15.41	22.69	1.82E−05	0.08
ENSG00000137070.11	IL11RA	8.39	12.94	1.99E−03	0.1
ENSG00000056736.5	IL17RB	5.51	12.06	2.28E−03	0.05
ENSG00000145623.7	OSMR	4.17	9.66	9.99E−05	0.06
ENSG00000115604.6	IL18R1	1.18	6.92	0.32	0.03
ENSG00000115594.7	IL1R1	4.19	6.52	4.06E−03	0.04
ENSG00000163702.14	IL17RC	4.51	6.39	2.10E−03	0.01
ENSG00000243646.3	IL10RB	2.6	4.01	0.04	0.01
ENSG00000131724.6	IL13RA1	2.97	3.39	1.39E−03	0.02
ENSG00000172458.4	IL17D	1.01	1.35	0.92	0.04
ENSG00000177663.8	IL17RA	−1.96	−2.6	0.01	0.01
ENSG00000240972.1	MIF	−2.73	−2.67	9.35E−04	1.25E−03
ENSG00000110944.4	IL23A	−8.56	−8.56	5.93E−03	5.93E−03
ENSG00000144730.12	IL17RD	−10.24	−10.24	4.19E−04	4.19E−04

**Table 2:** Differentially expressed genes in TNF and other inflammatory pathways.

**Feature ID**	**Gene ID**	**Fold change (Day 15 vs Day 5)**	**Fold change (Day 21 vs Day 5)**	***P*-value (Day 15 vs Day 5)**	***P*-value (Day 21 vs Day 5)**
**TNF ligands and receptors**					
ENSG00000121858.6	TNFSF10	4.06	11.4	0.04	0.05
ENSG00000239697.4	TNFSF12	11.82	7.99	2.85E−04	2.88E−03
ENSG00000183578.4	TNFAIP8L3	3.85	3.43	3.61E−03	2.13E−03
ENSG00000120889.8	TNFRSF10B	1.36	1.54	7.62E−05	2.90E−03
ENSG00000164761.4	TNFRSF11B	−3.21	−2.01	0.04	0.01
ENSG00000127863.11	TNFRSF19	−3.13	−3.91	3.62E−04	1.68E−03
ENSG00000215788.5	TNFRSF25	−3.41	−4.07	4.63E−03	9.28E−05
ENSG00000141655.9	TNFRSF11A	−6.81	−6.81	6.68E−06	6.68E−06
**Intra-cellular factors**					
ENSG00000196954.7	CASP4	7.07	17.6	0.14	0.05
ENSG00000156711.10	MAPK13	18.33	16.96	4.81E−05	2.07E−03
ENSG00000125347.8	IRF1	1.96	3.7	0.02	1.97E−03
ENSG00000064012.15	CASP8	3.99	3.28	8.92E−03	0.01
ENSG00000100030.9	MAPK1	3.76	2.48	2.52E−03	1.08E−03
ENSG00000109320.6	NFKB1	2.45	2.45	0.02	0.06
ENSG00000160703.9	NLRX1	2.55	2.27	0.05	0.04
ENSG00000138794.5	CASP6	1.85	2.14	0.02	6.67E−03
ENSG00000104825.11	NFKBIB	1.17	1.36	0.02	3.59E−04
ENSG00000168040.4	FADD	−1.48	−2.07	5.38E−03	1.62E−03
ENSG00000076604.8	TRAF4	−1.58	−2.08	0.02	8.68E−03
ENSG00000107643.10	MAPK8	−2.08	−2.09	0.01	9.92E−05
ENSG00000022556.10	NLRP2	−1.27	−3.58	3.55E−03	4.01E−03
ENSG00000127191.11	TRAF2	−4	−4.06	0.02	0.03
ENSG00000106144.14	CASP2	−4.14	−5.44	7.62E−05	3.13E−04
**HLA markers**					
ENSG00000198502.5	HLA-DRB5	14.6	6.46	7.39E−03	0.01
ENSG00000196126.6	HLA-DRB1	10.07	4.92	6.12E−03	0.01
ENSG00000204592.5	HLA-E	2.54	4.88	0.02	6.28E−03
ENSG00000204525.8	HLA-C	1.28	1.5	0.05	0.03
ENSG00000206503.6	HLA-A	−1.33	1.05	7.10E−03	0.64
ENSG00000234745.3	HLA-B	−3.2	−2.02	0.02	0.03
ENSG00000179344.11	HLA-DQB1	−2.25	−2.11	0.02	0.06

Upregulated genes are shaded in grey.
