# Supplementary material for: Profile of Inflammation-associated genes during Hepatic Differentiation of Human Pluripotent Stem Cells
Source: Data Brief. 2015 Nov 9;5:871–8. doi: 10.1016/j.dib.2015.10.023 (PMC4669432; doi:10.1016/j.dib.2015.10.023)
Supplement: Supplementary file 1 — Supplementary material [file mmc1.docx]

Conflict of interest

The authors declare no conflict of interest.
